# The Unique Extended Selection Cohorts Design for the Evaluation of the School-Based Jump-In Intervention on Dietary Habits: A Study Protocol

**DOI:** 10.3390/ijerph17041145

**Published:** 2020-02-11

**Authors:** Froukje E. Takens, Vincent Busch, Joanne K. Ujčič-Voortman, Manon van Eijsden, Mai J. M. Chinapaw

**Affiliations:** 1 Department of Public and Occupational Health, Amsterdam Public Health Research Institute, Amsterdam UMC, Vrije Universiteit Amsterdam, Van der Boechorststraat 7, 1081 BT Amsterdam, The Netherlands; m.chinapaw@amsterdamumc.nl; 2 Sarphati Amsterdam, Public Health Service of Amsterdam, Nieuwe Achtergracht 100, 1018 WT Amsterdam, The Netherlands; vbusch@ggd.amsterdam.nl (V.B.); jujcic@ggd.amsterdam.nl (J.K.U.-V.); 3 Voedingsjungle, p/a Arnhemseweg 114, 3817 CK Amersfoort, The Netherlands; manon@voedingsjungle.nl

**Keywords:** school-based intervention, dietary behaviour, children, study protocol, Extended Selection Cohorts design, mixed methods, effect evaluation, process evaluation

## Abstract

*Background*: To promote healthy dietary and physical activity behaviour among primary school children, the city of Amsterdam structurally implements the school-based Jump-in intervention in over half of its primary schools. Previously shown to be effective in stimulating physical activity and outside recess play, our study is the first to evaluate Jump-in’s effect on children’s dietary behaviour. Evaluating the effectiveness and implementation process of an intervention in a real-life setting requests an alternative study design. *Methods*: we chose a mixed-methods, quasi-experimental Extended Selection Cohorts design to evaluate Jump-in’s effectiveness and implementation process. Children and parents from the first ten primary schools that enrolled in the programme in 2016–2017 were invited to participate. The primary outcomes were children’s dietary behaviour and behavioural determinants, assessed by child and parent questionnaires, and photographs of the food and drinks children brought to school. Process indicators, contextual factors and satisfaction with the programme were assessed by interviews with health promotion professionals, school principals, school project coordinators, and teachers; focus group discussions with parents and children; and document analysis. *Discussion*: Conducting research in a real-life setting is accompanied by methodological challenges. Using an Extended Selection Cohorts design provides a valuable alternative when a Randomized Controlled design is not feasible.

## 1. Introduction

Childhood overweight and obesity are leading health concerns with a prevalence of 18 per cent of children 5 to 19 years old worldwide [1]. Although childhood overweight rates appear to stabilize in some high-income countries [2], overweight rates are still significantly higher and rising in lower socio-economic and migrant groups [3,4]. Childhood obesity increases the risk of early-onset physical health issues (e.g., type 2 diabetes, cardiovascular disease and some cancers) [5] as well as social and mental health issues (e.g., depression, being bullied and low self-esteem) [6]. Furthermore, it is likely to track into adulthood [7]. These health inequalities and adverse health outcomes highlight the importance of early targeted childhood obesity prevention.

A healthy diet is one of the key components in the prevention of overweight and obesity [5]. Yet, only about 40 per cent of children in The Netherlands meet daily fruit and vegetable intake recommendations [8] and roughly 90 per cent of Dutch children exceed the WHO’s daily recommendations of sugar intake [9,10] with sugar-sweetened beverages and unhealthy snacks being the biggest contributors [11].

School-based interventions may be promising with regard to stimulating fruit and vegetable intake among children [12]. An example of a school-based intervention that aims to promote children’s healthy dietary behaviour is a lunchbox intervention i.e., an intervention that aims to improve the foods and beverages children consume at school that are generally packed by parents. A recent meta-analysis reported an overview of lunchbox interventions [13] showing that lunchbox interventions increased the provision of vegetables, but the effects on fruit, discretionary foods and sugar-sweetened beverages were unclear. The authors recommend examining moderation of intervention effects by socio-economic position (SEP).

While dietary behaviour (e.g., drinking sugar-sweetened beverages) tends to be worse among children from ethnic minority groups [4,14] and people with a low SEP benefit more from healthy options in the food environment [15], effective interventions on dietary habits for children with a lower SEP or from an ethnic minority group are lacking [16]. To promote healthy dietary behaviour among children living in disadvantaged neighbourhoods, the Public Health Service of Amsterdam implemented an intervention targeting dietary habits in primary schools in disadvantaged neighbourhoods in Amsterdam [17,18]. In these neighbourhoods, obesity rates tend to be higher [19]. This intervention aims to implement a healthy nutrition school policy, including a healthy lunchbox intervention, and is part of the Jump-in programme that also focuses on promoting physical activity which has been evaluated previously [20,21]. Box 1 presents a detailed description of Jump-in.

Box 1 **What is the Jump-in intervention?**

*Multi-component intervention*. Jump-in is a multi-component primary school-based intervention that aims to stimulate physical activity [18,20,21], active school recess play [22] and healthy dietary habits in primary school children in Amsterdam, The Netherlands. Jump-in mainly focusses on Amsterdam’s areas where health inequalities prevail and where obesity rates are highest, which often coincides with a target population with a relatively high percentage of children with a low socio-economic position and/or from ethnic minority groups. In order to stimulate healthy dietary habits, a school-wide healthy school nutrition policy is implemented at primary schools, which means that for the morning break children exclusively drink water, tea without sugar or milk (1), and eat fruit and vegetables (2); for lunch children drink water, tea without sugar or milk (3), and eat whole wheat bread or products (4); and special treats (e.g., during birthday celebrations) comprise of small portion sizes, healthy foods or are non-food treats (5).

*Implementation strategies*. Jump-in comprises of a 3-year implementation process at schools, which is facilitated by a team of specialised health promotion professionals. After enrolment, the health promotion professional organises meetings with the school principal and the school’s Jump-in coordinator. The latter is a school employee who coordinates the implementation of the healthy nutrition policy and other parts of Jump-in. Together they formulate a tailored plan to fit the needs of the school, e.g., tailoring communication to different literacy levels and/or cultural backgrounds of children and parents who attend the school. The health promotion professional keeps structured and detailed logs of these meetings and implementation processes in a so-called Jump-in scan. Parental involvement is also an important aspect, because parents generally pack children’s lunchboxes and play an important role in children’s dietary habits at home. In order to stimulate teachers, parents and children to adhere to these outcomes, trained professionals such as dieticians provide workshops about corresponding themes (i.e., water, fruits and vegetables, breakfast and lunch, and treats). These professionals are trained to work with Jump-in’s target population. Posters and other communication and educational tools are provided to aid the embedding of the healthy nutrition policy in Jump-in schools. All programme components have been developed following the Intervention Mapping Protocol.

*Embedding in municipal policy*. The Jump-in programme is structurally embedded within the Amsterdam Healthy Weight Approach (AHWA): an integral preventive programme to reduce and prevent obesity in Amsterdam [17,23]. This programme aims to achieve a healthier weight for more children in Amsterdam by addressing multiple settings (e.g., schools, neighbourhoods, health care, etc.) and stakeholders (e.g., professionals, parents, etc.) involved with children in Amsterdam [23]. AHWA was recently recognized by the WHO as a good practice example of how health behaviour change can be structurally shaped involving the target group in an urban setting [24].

The Jump-in intervention has been sustainably implemented at a large-scale; currently nearly 130 primary schools with over 22,000 children in the past 17 years [17]. To thoroughly evaluate the intervention’s effect on dietary behaviour, an evaluation study has been set up. However, the large-scale implementation prohibited randomization or comparison with comparable control schools. Other challenges are the complex setting where the implementation is highly dependent on several stakeholders and the lack of accurate measures of children’s dietary behaviour. These challenges were tackled by using a mixed methods Extended Selection Cohorts (ESC) design.

This paper describes the design of a mixed methods effect and process evaluation study of a school-based intervention implemented in real-life. The aim of our study is two-fold: we evaluate the effects of Jump-in on dietary behaviour and behavioural determinants of children 4 to 12 years old and we evaluate the implementation process of the Jump-in programme in primary schools. Additionally, methodological challenges due to the real-life setting and proposed solutions are discussed.

## 2. Materials and Methods

### 2.1. Study Design

#### 2.1.1. Mixed Methods Design

We used quantitative methods to evaluate the effectiveness of the programme. Additionally, in the process evaluation, both quantitative and qualitative methods were used to monitor the implementation process and unravel potential underlying reasons for (in)effectiveness of the programme. Data collection took place between November 2016 and June 2019.

#### 2.1.2. Effect Evaluation

The Extended Selection Cohorts (ESC) design entails a cohort-longitudinal design with adjacent cohorts [25], that are measured at two or more time points [26]. Each cohort represents an age cohort including subjects of approximately the same age.

**Figure 1:**
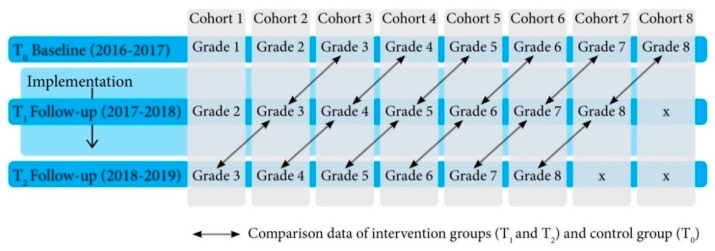
Extended Selection Cohorts design to evaluate the effectiveness of the intervention on dietary habits.

In our study, each cohort—represented by school grades—was measured three times: at baseline, 12 and 24 months after baseline measurements. Each cohort at follow-up was compared to the same-age adjacent baseline cohort group (see Figure 1). For instance, the measures of 2-year exposed cohort 3 (grade 5 in 2018–2019) were compared to the measures of 1-year exposed cohort 4 (grade 5 in 2017–2018) and to baseline measurements of cohort 5 (grade 5 in 2016–2017).

#### 2.1.3. Process Evaluation

**Figure 2:**
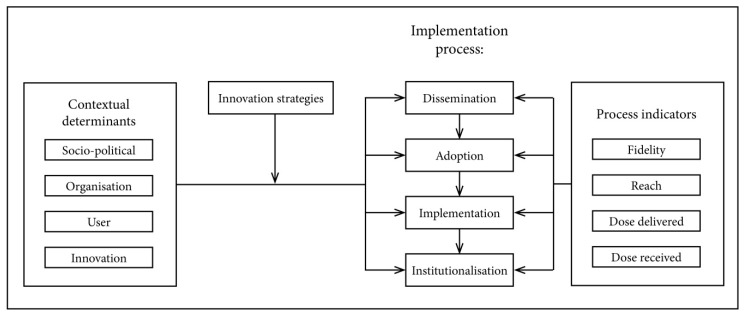
Framework representing the Jump-in intervention on dietary habits innovation process (adapted from Fleuren et al. [29]) and based on the Jump-in physical activity intervention evaluation [21].

The process evaluation focused on the implementation lessons learned and to better grasp how and under which conditions the intervention may be effective or not [27,28]. Figure 2 illustrates the process measurements.

#### 2.1.4. Quantitative Implementation Measures

Quantitative measures (i.e., interviews and document analyses) of fidelity, reach, dose delivered and dose received were assessed to capture the quantity and quality of Jump-in’s implementation [30,31]. Fidelity refers to the extent to which the intervention was delivered as intended (i.e., quality of the implementation). Reach is defined as the extent to which the target group participates in the intervention (e.g., proportion of teachers that carry out the healthy nutrition policy in their classroom). Dose delivered means the number of intended intervention units that have been provided to the participants (i.e., how many of the five components of the healthy nutrition policy have been implemented). Dose received refers to the extent to which participants actively engage with, interact with, are receptive to and/or use the intervention components (e.g., how did parents respond to specific activities) [32,33].

Qualitative contextual measures: qualitative methods (i.e., interviews, focus group discussions and document analyses) were used to explore how the context influenced implementation and outcomes; we studied external elements that may act as a barrier or facilitator to the implementation of the intervention on dietary habits. The implementation of the intervention on dietary habits took place in a real-life setting which allowed us to study whether context affects the outcome of the study [30], and hence, help interpret potential differences in outcomes between schools. Based on the framework of Fleuren et al. in the process of implementation there are several stages, which include the dissemination, adoption, implementation and continuation phases. When the implementation process transits from one phase to the next, it can be influenced by certain external contextual determinants [29]. These determinants were divided into four categories: characteristics of the socio-political context (e.g., fit with existing school legislation), characteristics of the organization (e.g., decision-making processes in the organization), characteristics of the user (e.g., knowledge of teachers) and characteristics of the innovation (e.g., complexity of the Jump-in programme). The relation between these determinants and the implementation process can be affected by innovation strategies; these strategies include ways to facilitate the implementation process such as promotional materials.

*Experience measures:* qualitative methods (i.e., interviews and focus group discussions) were used for the subjective evaluation (i.e., satisfaction) of the intervention on dietary habits.

### 2.2. Procedures

#### 2.2.1. Recruitment of Primary Schools

**Figure 3:**
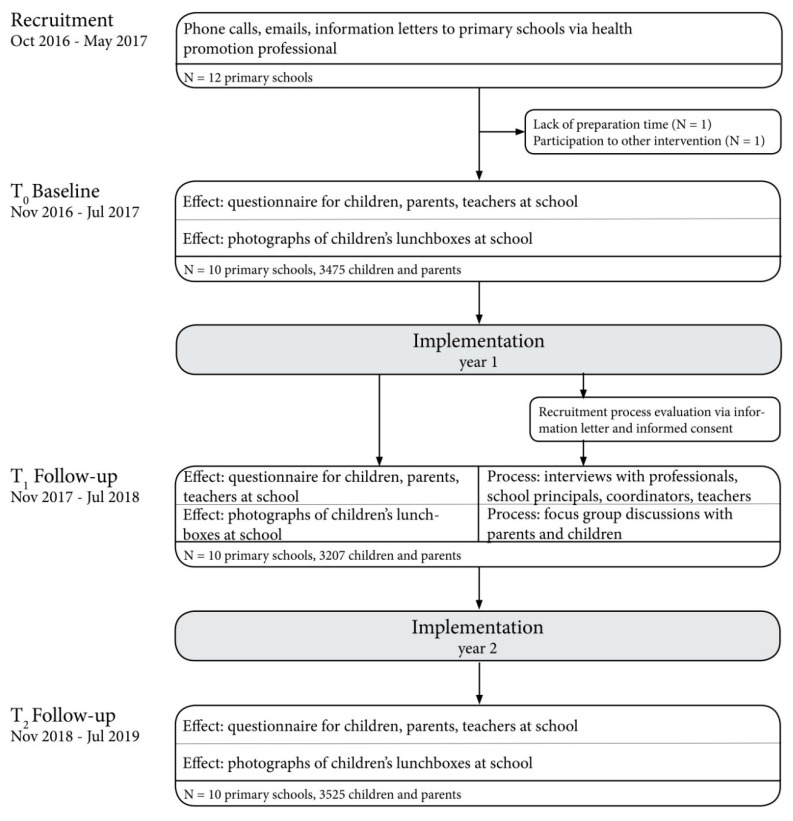
Flowchart of the study.

Figure 3 shows a flow chart that illustrates recruitment procedures. Schools that enrolled in the intervention on dietary habits in 2016–2017 were contacted to participate in the evaluation study. With the help of health promotion professionals, we contacted twelve primary schools in the city of Amsterdam that all agreed to participate. However, two schools were excluded due to logistical reasons (n = 1) or because the school also participated in comparable health promotion interventions (n = 1). Therefore, the final sample consisted of ten primary schools.

#### 2.2.2. Consent

Prior to data collection, parents received an information letter about the study and an opt-out option to withdraw their child from participation to the effect evaluation. Prior to the process evaluation, the parents of children in grades 5 through 8 received additional information on the process evaluation and were asked to give informed consent for their own or their child’s participation in a focus group discussion. School staff members received an information letter, accompanied by an informed consent form. The implementation of Jump-in in primary schools is part of the usual care of the Public Health Service of Amsterdam and the data was collected in accordance with organizational legislation. The research protocol was approved by the Medical Ethical committee of the VU University Medical Centre which concluded that it does not fall within the scope of the Medical Research Involving Human Subjects Act (study protocol 2016.415). Due to changed privacy legislation in The Netherlands during the study, as of May 2018 we removed children’s age from the questionnaires to protect their privacy. The trial was pre-registered (ISRCTN76414974).

### 2.3. Outcome Measures

#### 2.3.1. Effect Measures

The main outcome was children’s dietary behaviour at school; we examined differences in the consumption of water or tea without sugar, milk, sugar-sweetened beverages, fruits or vegetables, whole wheat bread, and unhealthy snacks. We included a-priori categories as an indicator of children’s dietary behaviour that were targeted by the intervention (i.e., the consumption of water or tea without sugar, milk, sugar-sweetened beverages, fruits or vegetables, whole wheat bread, and unhealthy snacks). Secondary outcome measures included dietary behaviour at home with regard to the products mentioned above and several determinants of dietary behaviour: attitude, knowledge, social norm, modelling, physical environment, liking and habit formation.

To assess children’s dietary behaviour, children, parents and teachers filled in questionnaires and we photographed the food and drinks children brought to school at T_0_, T_1_ and T_2_. The executive researcher and research assistant conducted the measurements with support from health promotion professionals and MSc students. In all grades children’s lunchboxes were photographed. In addition, children in grade 5–8 filled in questionnaires which was aided by the research team when a question was unclear. Data collection followed a few logistical considerations: (1) data collection was conducted before 10 AM (school breaks usually take place at 10 AM), (2) data collection was not conducted on Mondays in grades 5–8 (to avoid that children reported on weekend days), (3) data collection at fruit days was avoided (i.e., Dutch schools can sign up for the EU schoolfruit project in which they receive free fruits/vegetables a few days a week for 20 weeks), and (4) short schooldays were avoided. The children received a questionnaire for their parents in an envelope to take home. A few days before the deadline to hand in the questionnaire, the parents received a reminder to fill in the questionnaire. Prior to the start of Jump-in, health promotion professionals made agreements with schools ensuring the schools’ involvement with the programme and introduced the evaluation study. Subsequently, the coordinating researcher explained in detail what the study entailed including the required time investment. After each data collection wave schools received an incentive and a factsheet illustrating the percentages of drinks and foods consumed by the pupils. Additionally, the existing relationship between the health promotion professional and the school facilitated optimal compliance to the study protocol. Completed parent questionnaires were returned to the school and collected by the teacher. After data collection, the questionnaires were scanned. The food groups depicted on the photographs were entered in a Microsoft Access database by one the researchers. Categorising products to certain food groups and specifying the quantities was done based on an established Dutch Food Composition Database [34]. In the Netherlands, lunch boxes, packages and sipping cups have a standardized size, which gave us an indication of the quantity of the products consumed by children. Beverages are usually offered in 0.21 (e.g., packages of sugar-sweetened beverages), 0.2751 (e.g., sipping cups) or 0.51 (e.g., water bottles) packages. Bread usually has a standardised size as well, which enabled us to count the number of slices of bread. Portions of fruits and vegetables were counted based on the guidelines from the Dutch Food Composition Database. For instance, two mandarins are classified as one portion of fruit and five cherry tomatoes as one portion of vegetables. Last, we counted the number of snacks, which were usually packed per portion. After processing the photographs, a second researcher checked congruence. When dissonance between interpretation of food groups or quantities occurred, a third researcher checked these photographs and disagreement was discussed until consensus was reached.

*Questionnaires.* At-school and at-home dietary habits were assessed using questionnaires for parents and 8–12 year old children, and through short questionnaires for teachers. Questionnaires for parents and children comprised of three parts. First, the consumption of water or tea without sugar, milk, sugar-sweetened beverages, whole wheat bread, fruit, vegetables and snacks was asked using an adapted 24-h recall method. The parent questionnaire additionally comprised an adapted food frequency questionnaire (FFQ). Questions regarding the determinants of children’s dietary behaviour included attitude, social norm and modelling with regard to parents and peers, physical environment, liking and dietary habits, using 5-point Likert scales with smileys. These smileys were added to the scales because visual stimulation aids children’s motivation to fill in a questionnaire [35,36]. Third, socio-demographic characteristics of the target population were asked [37].

*Pre-test Questionnaires.* Prior to data collection, relevant questions from the validated ENERGY questionnaires were selected to assess the dietary habits regarding the dietary outcome measures [38,39]. The questionnaires were supplemented with relevant questions from the ProChildren study (assessing fruit and vegetable consumption) [40,41] and ChecKid [42]. If necessary, questions were slightly adapted in consultation with experts. Questionnaires were pretested among children and parents, and the order was based according to the moment of the day rather than by product group to increase the questionnaire’s comprehensibility. Then questionnaires were pre-tested at an after school-care facility to examine face validity with the target group (i.e., children and parents from families of ethnic minority groups and/or low SEP in disadvantaged areas of Amsterdam). This led to small adaptations to further improve the understanding of the questions and increasing the fun of filling out the questionnaire.

*Photographs.* To assess at-school dietary habits we photographed all food and drinks children brought to school. In addition, we noted on special cards what food and drinks children brought to school to prevent misclassification of products, for instance, in real-life it is easier to judge how many slices of bread were packed in a lunchbox compared to the photographs. Sometimes it was not possible to see through children’s cups or lunchbox, for instance, when bread was wrapped in tinfoil. Due to hygienic reasons we then asked children what was in their cup or lunchbox. To guarantee their privacy, we made sure children were not visible on the photographs. Before processing the data, we double-checked whether photographs were truly anonymous (e.g., no names written on cups), and if not, we covered these identifiers in the photographs.

#### 2.3.2. Process Measures

Data on process indicators, contextual measures and subjective experience measures were collected by interviews, focus group discussions, and document analysis.

We aimed to interview one school principal, one at-school Jump-in coordinator, two teachers, six to eight parents and six to eight children per school, as well as the health promotion professional supporting that school. The interviews were planned around the T_1_ measurements, approximately 12 months after the start of the implementation of the intervention on dietary habits. Interviews and focus group discussions were held in Dutch and done by two researchers. One of the researchers led the interview or focus group discussion, the other researcher kept track of time and took minutes. We aimed to interview a diverse population of teachers (i.e., based upon gender, grade they educate), parents (i.e., gender, age of child), and children (i.e., gender, age). In order to facilitate recruitment of parents and children for the process evaluation, the schools’ Jump-in coordinator advised which approach would suit best to optimise participation in focus group discussions. An example was planning the discussions on a Wednesday morning because more parents are free from work. When parents and children signed up for the focus group discussion, they received a confirmation by e-mail or phone. If we were not able to recruit enough participants for a focus group discussion, we conducted a group interview. If schools didn’t participate during the first follow-up measurement, they participated during the second follow-up measurement.

*Interviews.* Semi-structured interviews were held to assess the process indicators (e.g., reach), contextual factors and satisfaction with the programme. These interviews were held with the health promotion professional, school principal, at-school Jump-in coordinator and teachers. Measured items in the interview guides were based on the topic lists used in the Jump-in physical activity study and DOiT study [21,43] and was built up according to the implementation process from Fleuren et al. [29]. An example of a question regarding process indicators included: “How many components of the Jump-in healthy nutrition policy have been implemented in this schools’ nutrition policy?” Examples regarding the implementation process were: “How and who decided to participate in the Jump-in programme?”, “Which factors hampered the implementation of the healthy nutrition policy at this school?” and “which factors hamper or facilitate a long-term implementation of this healthy nutrition policy at this school?” An example of a question regarding the innovation strategies was: “How many workshops took place?”

*Focus group discussions.* Focus group discussions were held to assess the contextual factors regarding the user and socio-political characteristics, innovation strategies and satisfaction with the programme. We conducted focus group discussions with parents and children. Items in the checklist were based on De Meij et al. [21] and van Nassau et al. [43]. In the focus group discussion with parents, we used different statements and sticky notes to discuss characteristics of the socio-political context (e.g., financial consequences of the intervention), innovation context (e.g., clarity of procedures), and innovation strategies (e.g., communication regarding the intervention). In the focus group discussion with children we used drawings and sticky notes to discuss the characteristics of the socio-political context (e.g., norm at home), the innovation context (e.g., clarity of procedures), and the innovation strategies (e.g., workshops).

*Document analysis*. Document analysis was included to assess process indicators, contextual factors and satisfaction. These documents comprised of Jump-in scan logs of effect data collection and interview notes. Jump-in scans are structured, uniform logs kept by the health promotion professionals to keep track of when which intervention components are implemented. It serves as an instrument to structure and aid the quality of intervention delivery and implementation. Furthermore, the logs of the effect evaluation data collection allowed us to monitor potential effect evaluation measurement flaws.

### 2.4. Analyses

#### 2.4.1. Quantitative Analysis

Prior to data collection, we estimated that 107 subjects for each cohort was needed (d = 0.5, SD = 1.0, α = 0.05, power 80%) in order to detect a statistical difference between our control (i.e., T_0_) and intervention groups (i.e., T_1_ and T_2_). Assuming each class consisted of a minimum of 25 students, five schools were required. When we took into account the cluster design and possible non-response, we took a sample that consisted of twice the number of schools needed [44]; hence, the sample size was ten primary schools.

We will conduct a series of multilevel regression analyses. This analysis takes into account different clusters (i.e., class level, potentially school level). Same-age data from different time points and cohorts will be compared, in which cohorts consist of a group of children from the same age from all ten participating schools. We will create a variable for at-school consumption by combining the food and drinks consumed in the first and second school break, i.e., sugar-sweetened beverages, water or tea without sugar, milk, whole wheat bread, fruit, vegetables, snacks consumed at school. This will be done based on the parent and child questionnaires, and the photographs. Behavioural determinants (attitude, knowledge, social norm, modelling, physical environment, liking and habit formation) will be derived from the parent and child questionnaires.

To analyse the effect of the intervention on the combined scores of at-school dietary behaviour and behavioural determinants, we will use multilevel regression analyses. For continuous outcome variables we will use linear multilevel regression models with time point as first level variable and grade as second level variable. When variables are not normally distributed, we will use Poisson multilevel analysis. For categorical variables we will use logistic multilevel models. To examine whether observations cluster at school level, we will add schools as third level in the linear models. If this is the case, schools will be added to all models—creating a three-level structure—and for categorical variables we will shift to a GEE analysis.

To explore the moderation effects by SEP, age in years, sex and ethnicity, we will add interaction terms to the model. When these terms are significant, we will stratify the analyses for these specific subgroups.

Finally, to study whether children compensate for at-school dietary changes when they are home, we will adjust at-school child dietary behaviour for at-home child dietary behaviour (i.e., combined score of consumption in the morning and after school, derived from the parent and child questionnaire). A significance level of *p* = 0.05 will be used and *p* = 0.10 for moderation effects. For the analyses we will use IBM SPSS Statistics Version 21.0 (IBM, Armonk, NY, USA) and STATA/IC 15.1 (StataCorp LLC., College Station, TX, USA).

#### 2.4.2. Qualitative Analysis

The semi-structured interviews and focus group discussions were recorded and transcribed. These transcriptions, the interview notes, logs, Jump-in scans will be analysed using MAXQDA 2018. We will conduct a content analysis in which we use the framework of Fleuren et al. [29] (Figure 2). The analysis will be an iterative process and will be conducted by two independent researchers (F.E.T. and S.J., V.T., V.D. or L.B., see Acknowledgements). At first, the researchers will do the analysis independently for all interviews at two schools. Then, the codes will be compared, and consensus will be reached, and one of the researchers then will code all the interviews.

## 3. Discussion

The purpose of this paper is to describe the mixed methods Extended Selection Cohorts design for the evaluation of an intervention targeting dietary habits, the methodical challenges due to the real-life setting and proposed solutions. This design enables us to collect valuable data on (1) the effects of a widely-disseminated intervention with a reach of over 22,000 children [17], (2) outcomes of children’s dietary behaviour reported by both parents and children (3) children’s dietary behaviour using self-reported (i.e., questionnaire) and objective measurements (i.e., photographs) and (4) the context, which helps interpretation of the outcomes. We chose this evaluation design because recruitment of comparable control schools and randomization was impossible. Due to the large-scale implementation of Jump-in in Amsterdam, finding a control group was virtually impossible. Furthermore, primary schools that enrolled in the Jump-in programme were predetermined. This prohibited random assignment to either an intervention or control group. We tackle this challenge by applying the quasi-experimental Extended Selection Cohorts design.

The ESC design is suitable in real-life settings in which the recruitment of control groups fails or intervention groups are predetermined, as it still allows comparison of same-age treatment groups [26]. Whereas in a standard Randomized Controlled Trial design, the intervention is randomly allocated to different schools; in the ESC design intervention and control groups belong to the same schools, and hence, fewer schools are needed. Since intervention and control groups belong to the same units and cohorts are represented by different grades, groups are recruited from a relatively stable population [26]. In many quasi-experimental designs treatment and control groups differ in socio-demographic characteristics which may be hard to monitor [26]. However, since in the ESC design groups originate from the same schools, groups are comparable in baseline characteristics (e.g., in age, SES and ethnicity). This is important in our setting, because dietary behaviour is likely different between different SES and ethnicity groups [4,45].

A limitation of the ESC design is the difference in the time points of data collection of control and intervention groups, since baseline measurements represent the control and the follow-up measurements represent the intervention groups. Therefore, the design does not allow to control for general societal time trends that coincide with the intervention, such as a new supermarket next to the school. To mitigate this limitation, we conduct an extensive process evaluation.

This evaluation study is set up in a complex real-life setting, which may influence how the intervention is delivered [27]. Contextual factors to consider are: the wide dissemination among primary schools in Amsterdam, the support of a team of health promotion professionals, and the embedding in health promotion programmes and Amsterdam’s municipality (i.e., ensuring structural, financial and political support and connections with other health promotion interventions in Amsterdam) [17]. These factors might influence the implementation and, thereby, the outcomes.

To get a grip on the complexity of the setting, we include a process evaluation. Process measures can be used to explain differences in effectiveness between clusters (e.g., schools), and understand how and under which conditions an intervention is effective [27,28].

Accurate measurement of dietary behaviour, especially in children, is challenging. In general, the validity of self-report is questionable, because children have a short attention span [35] and outcomes can be biased due to recall and social desirability [46,47,48,49]. For instance, children tend to overestimate their fruit and vegetable intake in self-reported dietary methods [50,51].

Another common challenge is to motivate people to participate and repeatedly fill out long questionnaires. In our study, data collection relied on a number of stakeholders to motivate children and parents to participate; we needed the help of the health promotion professional to contact primary schools, the school principal and Jump-in coordinator to approve study participation; lastly, the teachers to motivate children and parents to fill in questionnaires and allow us to photograph the food and drinks that children bring to school. Even though we incorporated strategies to optimise participation in this study (e.g., tailoring recruitment procedures to the schools’ needs), this remains challenging.

To address both these issues, we included photographs of children’s at-school food and drinks. This method is not prone to self-reported recall bias, and—compared to questionnaires—is less time consuming, labour intensive and costly. However, it can be questioned whether it is prone to social desirability, e.g., when children hide unhealthy foods or drinks during data collection, or are not honest about the content of covered products (i.e., due to hygiene reasons we did not unfold products wrapped in tinfoil).

Furthermore, this method measures the products children bring to school without knowing whether they actually consume it. Photographs might not be suitable to accurately assess quantity. However, in the present study, we were specifically interested in whether the intervention enabled a shift towards healthier alternatives (e.g., a shift from sugar-sweetened beverages to water). For this objective, photographs can be useful.

Although not extensively researched yet, some authors found that digital imaging is a reliable alternative method for measuring fruit and vegetable intake during school lunch [52]. The use of both questionnaires and photographs to measure dietary intake in our research allows us to assess the effect of the intervention and compare both methods.

## 4. Conclusions

We aimed to provide an example of using a mixed-methods, quasi-experimental Extended Selection Cohorts design to evaluate the effectiveness of a school-based intervention on children’s dietary habits as well as the implementation process in real-life.
